# QTL Mapping-Based Identification of Visceral White-Nodules Disease Resistance Genes in *Larimichthys polyactis*

**DOI:** 10.3390/ijms252010872

**Published:** 2024-10-10

**Authors:** Qian Li, Jiajie Zhu, Sifang Liu, Haowen Liu, Tianle Zhang, Ting Ye, Bao Lou, Feng Liu

**Affiliations:** 1 National Engineering Research Center for Marine Aquaculture, Zhejiang Ocean University, Zhoushan 316022, China; 15769214836@163.com; 2 Zhejiang Key Laboratory of Coastal Biological Germplasm Resources Conservation and Utilization, Institute of Hydrobiology, Zhejiang Academy of Agricultural Sciences, Hangzhou 310021, China; zhujiajie1225@163.com (J.Z.); lsf19971009@163.com (S.L.); liuhaowendandan@163.com (H.L.); 18252485520@163.com (T.Z.); 15tye@stu.edu.cn (T.Y.); 3 Key Laboratory of Applied Marine Biotechnology by the Ministry of Education, School of Marine Sciences, Ningbo University, Ningbo 315211, China; 4 College of Life Sciences, China Jiliang University, Hangzhou 310018, China

**Keywords:** *Pseudomonas plecoglossicida*, *Larimichthys polyactis*, genetic linkage map, QTL mapping, disease resistance

## Abstract

Disease outbreaks in aquaculture have recently intensified. In particular, visceral white-nodules disease, caused by *Pseudomonas plecoglossicida*, has severely hindered the small yellow croaker (*Larimichthys polyactis*) aquaculture industry. However, research on this disease is limited. To address this gap, the present study employed a 100K SNP chip to genotype individuals from an F1 full-sib family, identify single nucleotide polymorphisms (SNPs), and construct a genetic linkage map for this species. A high-density genetic linkage map spanning a total length of 1395.72 cM with an average interval of 0.08 cM distributed across 24 linkage groups was obtained. Employing post-infection survival time as an indicator of disease resistance, 13 disease resistance-related quantitative trait loci (QTLs) were detected, and these regions included 169 genes. Functional enrichment analyses pinpointed 11 candidate disease resistance-related genes. RT-qPCR analysis revealed that the genes of *chmp1a* and *arg1* are significantly differentially expressed in response to *P. plecoglossicida* infection in spleen and liver tissues, indicating their pivotal functions in disease resistance. In summary, in addition to successfully constructing a high-density genetic linkage map, this study reports the first QTL mapping for visceral white-nodules disease resistance. These results provide insight into the intricate molecular mechanisms underlying disease resistance in the small yellow croaker.

## 1. Introduction

The small yellow croaker (*Larimichthys polyactis*), a member of the order Perciformes and family Sciaenidae, is a warm–temperate demersal fish that inhabits the northwest Pacific Ocean, including the Bohai Sea, Yellow Sea, and East China Sea [1]. As one of the “Four Major Marine Products” in China, this fish is highly prized for its nutritional value, exquisite flavor, and delicate texture, making it a favored choice among consumers and a pivotal economic species with considerable aquaculture potential [2]. Significant advances in small yellow croaker aquaculture have recently been achieved, including full artificial breeding and large-scale cage farming [3], particularly along the coastal regions of the Zhejiang and Jiangsu provinces in China [4]. This transformation has positioned the small yellow croaker as a promising species for aquaculture. However, with the development of intensive farming models, the aquaculture environment has deteriorated continually, resulting in frequent disease outbreaks, which pose a formidable challenge to the sustainable development of the small yellow croaker aquaculture industry [5].

The etiology of diseases in marine aquaculture fish primarily includes pathogenic microorganisms (e.g., viruses, bacteria), parasites, and abiotic factors [6]. Among these, visceral white-nodules disease (VWND) caused by *P. plecoglossicida* has emerged as one of the most significant diseases in recent years [7]. Characterized by its swift onset and high infectivity, this disease has been documented in a range of fish species, including the orange-spotted grouper (*Epinephelus coioides*) [8], rainbow trout (*Oncorhynchus mykiss*) [9], crucian carp (*Carassius auratus*) [10], and large yellow croaker (*Larimichthys crocea*) [11]. The mortality rate due to this disease skyrocketed to 70–80%, leading to substantial economic losses in major cultures [12]. To address this pressing issue, the breeding of new disease-resistant strains with superior traits has become imperative. Marker-assisted selection (MAS), as opposed to traditional breeding methods, which are time-consuming, labor-intensive, and inefficient, is a more accurate, stable, and effective strategy [13]. MAS can shorten the breeding cycle significantly [14], ensuring the rapid development of resilient fish strains capable of thriving in challenging aquaculture environments.

With unprecedented advancements in high-throughput sequencing technology, SNP markers, characterized by their abundance and extensive distribution across the genome, have emerged as indispensable molecular tools [15,16]. They are widely used in genetic map construction, QTL mapping, and the identification of genetic resources [17]. SNP markers have been used to construct high-density linkage maps for diverse aquaculture species, such as the Atlantic salmon (*Salmo salar*) [18], catfish (*Silurus asotus*) [19], Japanese flounder (*Paralichthys olivaceus*) [20], Asian sea bass (*Lates calcarifer*) [21], Pacific oyster (*Crassostrea gigas*) [22], and large yellow croaker (*Larimichthys crocea*) [23]. Moreover, the utilization of high-density genetic markers for quantitative trait locus (QTL) mapping in aquaculture species represents a pivotal strategy in unraveling trait structures and pinpointing genomic regions, underlying genes, and causal mutations that govern phenotypic variability [24]. This approach has been implemented extensively to explore QTLs associated with disease resistance in aquaculture species, such as the red sea bream (*Pagrus major*) [25], Pacific oyster (*Crassostrea gigas*) [26], black rockfish (*Sebastes schlegelii*) [27], and turbot (*Scophthalmus maximus*) [28].

Understanding the key genes related to disease resistance is crucial for MAS and the healthy development of the aquaculture industry. Various disease resistance-related genes in aquaculture species have been identified. For example, QTL mapping in the three-spined stickleback (*Gasterosteus aculeatus*) revealed two genomic regions associated with exacerbated gut inflammation, unveiling candidate genes implicated in the coagulation/complement system, NF-κB and MAPK signaling pathways, as well as genes associated with intestinal and nervous system diseases [29]. In the Asian sea bass, QTLs and genes associated with resistance to the Singapore grouper iridovirus were identified [30]. However, little research has focused on QTLs and the functional genes related to VWND resistance in the small yellow croaker. Therefore, in the present study, individuals from an F1 full-sib family of small yellow croaker were evaluated using a 100K SNP chip for individual SNP genotyping and the construction of a high-density genetic linkage map. We performed QTL mapping specific to the VWND resistance trait to screen for disease resistance-related SNPs and identified functional genes associated with disease resistance. This groundbreaking research serves as a cornerstone in deciphering the intricate molecular mechanisms underlying disease resistance in the small yellow croaker as well as in other aquaculture animals, thereby providing invaluable insights for the sustainable development of the aquaculture industry.

## 2. Results

### 2.1. Genotyping and Genetic Marker Development

A total of 121 F1 offspring and two parents were tested using the 100K SNP chip, yielding 97,537 SNPs. After filtering, 74,097 SNP markers were obtained, among which 48,853 markers were available for the F1 population. Following filtering for segregation distortion, 43,765 SNPs were retained (Table 1). Through the process of binning, 18,036 bin markers were identified for subsequent analyses.

### 2.2. Construction of Linkage Maps

**Figure 1:**
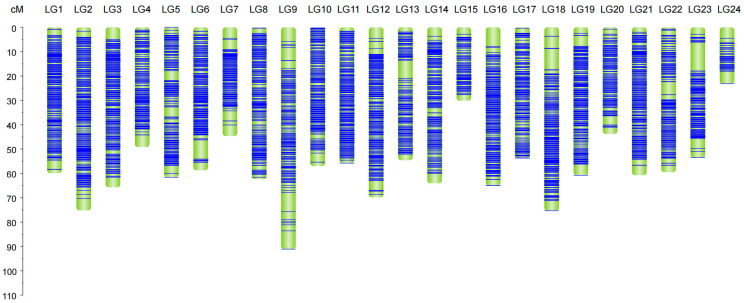
Genetic linkage map. Length and marker distribution of 24 linkage groups (LGs) in the bin map. The ordinate indicates the genetic distance. The abscissa indicates the linkage groups. Green represents the chromosome, and blue represents the bin markers and their genetic distance on the linkage groups.

Linkage maps with 24 linkage groups were constructed using the Perl SVG module based on the high-quality genetic markers. The male map, consisting of 12,108 bin markers, exhibited a total genetic distance of 1433.12 cM, averaging 0.12 cM per marker and featuring a maximum interval of 16.26 cM (Supplementary Table S1). The female map included 11,734 bin markers, spanning a total genetic distance of 1374.35 cM, also averaging 0.12 cM but with a maximum interval of 19.05 cM (Supplementary Table S2). The integrated map contained 17,735 bin markers, spanning a total genetic distance of 1395.72 cM, with an average distance of 0.08 cM and a maximum interval of 11.79 cM (Figure 1; Table 2).

### 2.3. QTL Mapping of Disease-Related Traits

**Figure 2:**
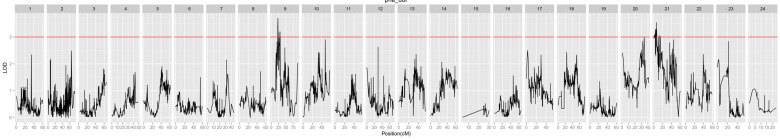
Mapping of disease resistance-related QTLs. The horizontal axis at the top indicates linkage group numbers. The horizontal axis at the bottom indicates the genetic distance for each linkage group. The vertical axis represents LOD values. The red line indicates the determined LOD threshold (=3).

A QTL analysis of SNP loci associated with the phe_dur phenotype was performed using the MQM mapping approach within the MapQTL6.0 software. At LOD = 3, 13 QTLs related to disease resistance were identified, distributed across two linkage groups designated LG9 and LG21 (Table 3, Figure 2). Specifically, LG9 contained four QTLs, each explaining 11% to 13.3% of the phenotypic variation. Among these, the QTL with the maximum LOD score of 3.71 explained 13.3% of the phenotypic variation, while the QTL with the minimum LOD score of 3.03 accounted for 11% of the phenotypic variation. LG21 contained nine QTLs, collectively explaining phenotypic variation within a range of 10.9% to 13.3%. Here, the QTL displaying the maximum LOD score of 3.53 contributed 12.7% to the phenotypic variation, and the QTL with the minimum LOD score of 3.01 explained 10.9% of the phenotypic variation.

### 2.4. Functional Enrichment Analysis of Candidate Genes

**Figure 3:**
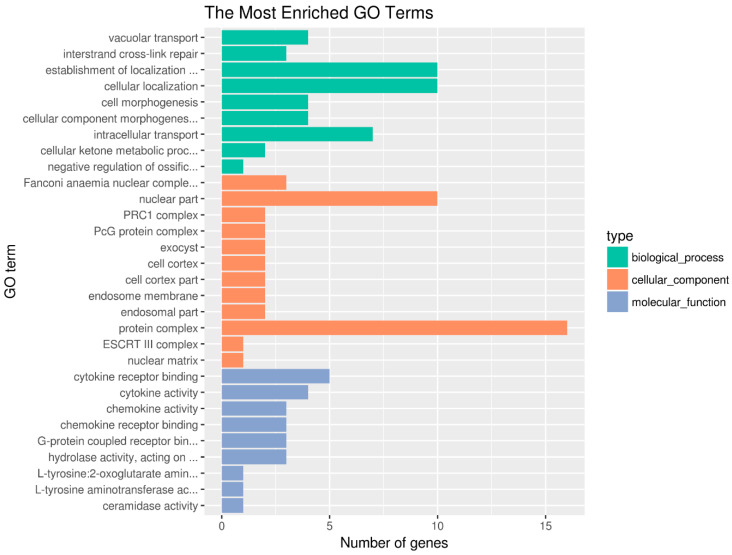
GO functional enrichment analysis for the genes. Top 30 significant enriched GO terms. Most of the genes were significantly assigned to the category of the protein complex, nuclear part, establishment of localization in the cell, cellular localization, intracellular transport, cytokine receptor binding, and so on.

Candidate genes were retrieved based on the annotated genome of the small yellow croaker. Within the 13 QTLs analyzed, 169 candidate genes were identified (Supplementary Table S3). Functional enrichment analyses of these candidate genes using the GO database revealed that the genes are mainly related to the terms of cellular localization, protein complex, chemokine activity, cytokine receptor binding, and several other terms (Figure 3; Supplementary Table S4). These results suggest that diverse biological processes contribute to resistance to VWND.

**Figure 4:**
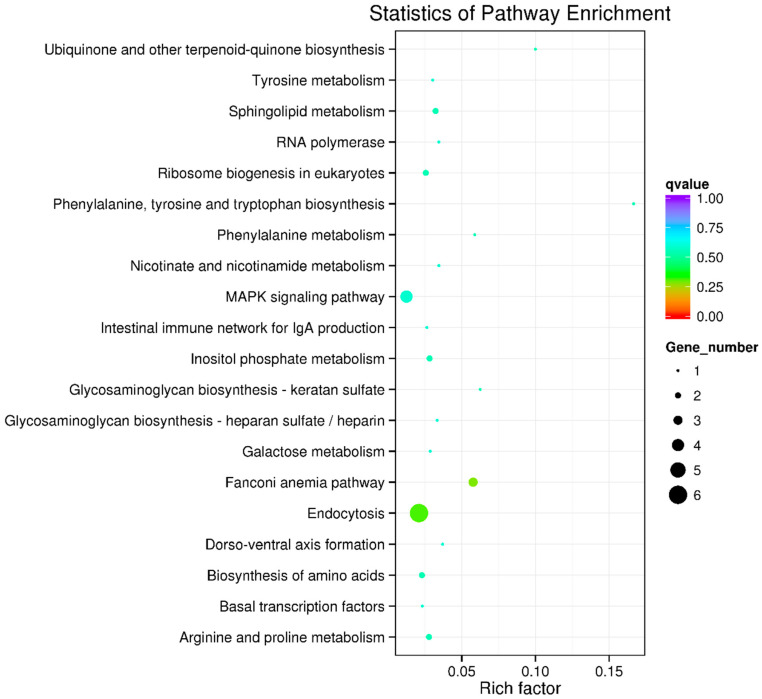
KEGG functional enrichment analysis for genes. The top 20 significant enriched pathways are shown, in which the most enriched pathways included endocytosis, the MAPK signaling pathway, the Fanconi anemia pathway, the biosynthesis of amino acids, sphingolipid metabolism, inositol phosphate metabolism, arginine and proline metabolism, and so on.

In the KEGG pathway enrichment analysis of the candidate genes, the top 20 enriched pathways were mostly related to cell growth, proliferation, metabolism, and apoptosis. These pathways encompass a range of biological processes, including endocytosis, the MAPK signaling pathway, the Fanconi anemia pathway, the biosynthesis of amino acids, sphingolipid metabolism, inositol phosphate metabolism, arginine and proline metabolism, ribosome biogenesis in eukaryotes, the intestinal immune network for IgA production, and several other metabolic pathways (Figure 4; Supplementary Table S5). These findings suggest that the response to pathogen stimulation involves both metabolic processes and the immune system.

### 2.5. Identification of Disease Resistance Gene Candidates

**Figure 5:**
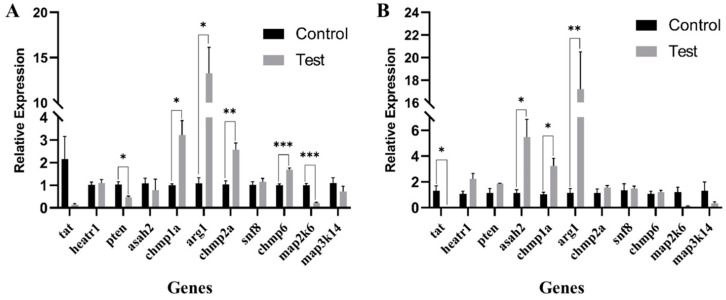
Expression levels in spleen (**A**) and liver (**B**) tissues. In the test group, fish were injected with *P. plecoglossicida* for 72 h; in the control group, fish were injected with TSB solution for 72 h. In spleen tissue, *pten, chmp1a*, *arg1*, *chmp2a*, *chmp6*, and *map2k6* levels differed significantly between groups. In liver tissue, *tat*, *asah2*, *chmp1a*, and *arg1* levels differed significantly between groups. * *p* ≤ 0.05; ** *p* ≤ 0.01; *** *p* ≤ 0.001.

A total of 11 candidate genes were evaluated using RT-qPCR; these included six genes associated with the most enriched pathways of endocytosis and the MAPK signaling pathway, as well as five additional randomly selected potential genes related to disease resistance obtained from the present study (Table 4). The results revealed significant differences in the expression levels of eight genes in spleen and liver tissues when compared with levels in the control group. In spleen tissue, the expression levels of *pten*, *chmp1a*, *arg1*, *chmp2a*, *chmp6*, and *map2k6* differed significantly, with *chmp1a*, *arg1*, *chmp2a*, and *chmp6* exhibiting upregulation and *pten* and *map2k6* displaying downregulation in the test group (Figure 5A). In liver tissue, a pattern of differential expression was observed for *tat*, *asah2*, *chmp1a*, and *arg1*, with *asah2*, *chmp1a*, and *arg1* showing upregulation and *tat* showing downregulation in the test group (Figure 5B). Notably, *chmp1a* and *arg1* demonstrated significant differential expression in both spleen and liver tissues, which were consistently upregulated.

## 3. Discussion

MAS is an efficient tool for genetic improvement, leveraging the intimate link between molecular markers and genes that dictate targeted traits. Molecular markers provide a basis for evaluating the presence of a target gene and thereby for the selection of favorable traits [31]. High-quality genetic linkage maps serve as vital tools for QTL mapping, MAS, and genetic improvement in many aquaculture species [32]. For instance, a genetic linkage map has been constructed for black carp (*Mylopharyngodon piceus*) using 128 F1 progeny and 10,390 SNPs, spanning a total length of 1708.53 cM with an average genetic distance of 0.51 cM [33]. Similarly, a high-density genetic linkage map for the Japanese flounder was constructed using 13,362 SNPs, with a total length of 3497.29 cM and an average interval of 0.47 cM [20]. For grass carp (*Ctenopharyngodon idella*), a genetic map was constructed with 3979 SNPs, with a total length of 1752.742 cM and an average marker interval of 0.44 cM [34]. In the present study, aiming for more accurate QTL mapping with narrower intervals, a high-density genetic linkage map was constructed based on 18,036 bin markers. The total map length was 1395.72 cM, with an average interval of 0.08 cM, ensuring the provision of accurate physical locations. The differences in map length and precision compared with those of previous studies may be attributed to the genetic variation resulting from different mapping populations.

Disease has been a pivotal trait targeted by aquaculture breeders for decades, and it represents the single most significant contributor to economic losses within the aquaculture industry [35]. In recent years, with the successful construction of high-density genetic linkage maps, extensive research has focused on QTLs related to disease resistance traits. In a study involving 520 Asian sea bass (*Lates calcarifer*), 23 QTLs associated with resistance to viral nervous necrosis were detected, explaining 2.2–4.1% of the phenotypic variation [36]. In another study, QTL mapping for resistance to the Singapore grouper iridovirus in 2000 Asian sea bass identified four QTLs on four linkage groups, explaining 7.5–15.6% of the phenotypic variation [30]. In the present study, QTL mapping identified 13 QTLs related to resistance to VWND in the small yellow croaker, explaining 10.9–13.3% of the phenotypic variation. Compared with QTLs associated with resistance to *Edwardsiella tarda* in the Japanese flounder, which explained 16.0–89.5% of the phenotypic variation with a similar sample size of 106 fish [37], the proportion of variance explained in this study was relatively smaller. This difference may be due to the complexity of disease resistance traits in fish, which are influenced by species, the external environment, sample size, and other factors.

Upon the infection of fish by pathogenic bacteria, a complex series of immune defense mechanisms are promptly activated. The immune response consumes considerable energy, necessitating metabolic adjustments to redirect energy resources [38]. In this process, many immune processes related to carbohydrate, lipid, and amino acid metabolism are activated [39,40]. Based on the reference genome annotation, candidate genes were obtained. Functional enrichment revealed that these genes are involved in immune pathways, such as “Endocytosis”, “MAPK signaling pathway”, “Fanconi anemia pathway”, “phenylalanine, tyrosine, and tryptophan biosynthesis”, and “intestinal immune network for IgA production”. To validate our findings, the expression levels of 11 genes (6 in the “endocytosis”, and “MAPK signaling pathway” and 5 potential disease resistance-related genes), were evaluated in the spleen and liver tissues of fish subjected to pathogen infection. Among the 11 genes, 6 showed significant differential expression only in the spleen, 2 showed significant differential expression only in the liver, and 2 (i.e., *chmp1a* and *arg1*) were significantly differentially expressed in both organs under pathogen infection. These results indicate that *chmp1a* and *arg1* as crucial candidate genes associated with resistance to VWND. These findings provide valuable insights into the genetic underpinnings of disease resistance in fish and offer potential targets for future breeding and genetic improvement programs.

Among the candidate genes, *arg1* (Arginase 1) is a key molecule involved in arginine metabolism. Arginine can enhance antioxidant defense in fish, mitigating inflammatory responses [41,42]. *Arg1* metabolizes L-arginine into urea and L-ornithine, which subsequently yield proline and polyamines vital for cell proliferation and collagen synthesis [43]. *Arg1* protects against inflammation, tumor immunity, fibrosis, and immunosuppression-related diseases mainly via its regulatory effects on L-arginine metabolism in immune cells, including macrophages [44,45]. Therefore, it can be inferred that in response to pathogen infection, fish exhibit an inflammatory response, prompting *arg1* upregulation to protect against tissue damage caused by inflammation [46]. *Chmp1a*, *chmp6*, and *chmp2a* are members of the ESCRT-III family, which is crucial for cellular functions from division to endosome maturation [47,48]. ESCRT-III proteins mediate membrane remodeling in multivesicular body formation, cytokinesis, and virus release [49]. They also restrict pro-inflammatory responses post-inflammasome activation [50]. *Chmp1a* modulates apoptosis via Bax/Bcl-2 and MDM2/p53, influencing proliferation and inhibiting differentiation [51,52]. Its dysfunction disrupts ESCRT-III-mediated vesicle release, hindering brain progenitor proliferation [53]. *Chmp1a* overexpression using plasmids inhibits renal tumor growth in vitro and in vivo [54]. As an autophagosome regulator, the depletion of *chmp2a* triggers a caspase-8 cascade, inducing apoptosis in osteosarcoma and neuroblastoma cells [55]. *Chmp2a* and tumor-EVs can induce NK cell apoptosis, limiting cytotoxicity [56]. Overexpression of *chmp6* promotes apoptosis-related cell death, suggesting its role in tumorigenesis and apoptosis [57]. Blocking the ESCRT-II-CHMP6 interaction disrupts cytokine shedding, leading to cell death [58]. Thus, *chmp1a*, *chmp6*, and *chmp2a* may regulate apoptosis and proliferation in response to pathogen stress.

The *map2k6* (mitogen-activated protein kinase kinase 6) gene belongs to the MAPK (mitogen-activated protein kinase) family. MAPK kinases facilitate protein phosphorylation, forming complex regulatory networks that control gene expression and play crucial roles in cell proliferation, apoptosis, immune defense, and humoral immunity [59]. Extensive research has focused on their roles in various aquaculture species. For instance, chlorpyrifos activates the MAPK signaling pathway, resulting in necrotic cell death and inflammatory damage [60,61]. Conversely, downregulating the MAPK signaling pathway can mitigate the inflammation induced by polystyrene-MP infection in the head kidney cells of Nile tilapia (*Oreochromis niloticus*) [62]. Additionally, alkaline exposure disrupts ammonia metabolism and ammonia accumulation in the Chinese mitten crab, activating the MAPK signaling pathway and ultimately leading to apoptosis [63]. *Map2k6*, plays a central role in regulating cell functions [64]; specifically, it promotes cell proliferation [65], as evidenced by its essentiality in esophageal adenocarcinoma cell proliferation [66]. Similarly, downregulation of *map2k6* inhibits the MAPK pathway, reducing the proliferation and invasion of cervical cancer cells [67]. In the present study, *map2k6* was downregulated in fish subjected to the pathogen infection. This response inhibits the proliferation of infected cells, bolstering the immune response and minimizing tissue damage.

*Pten* (phosphatase and tensin homolog) is a phosphatase that inhibits the PI3K/AKT signaling pathway and regulates autophagy, apoptosis, and cell proliferation [68]. Its suppression activates the PI3K/AKT signaling pathway, promoting gastric cancer cell growth, the epithelial–mesenchymal transition, and metastasis [69]. In the Japanese flounder, *pten* and its regulatory miRNAs modulate autophagic cell activation via the AKT/mTOR pathway during bacterial and viral infections, inducing apoptosis [70]. Additionally, *pten* acts as an immune modulator in inflammatory responses [71,72]. Therefore, *pten* may be involved in inflammatory responses and apoptosis under pathogenic attacks in fish. *Asah2* encodes N-acylsphingosine amidohydrolase 2 and is a key neutral ceramidase, instrumental in preventing inflammation [73]. This enzyme catalyzes the hydrolysis of ceramide, which is a molecule that modulates cellular stress responses, directs sphingolipid metabolism, and regulates cell apoptosis and aging [74,75]. *Asah2* exerts protective effects by destabilizing the p53 (tumor suppressor) protein, thereby inhibiting the p53 pathway and protecting against ferroptosis [76]. Furthermore, *asah2* has been identified as a crucial target of Fut2 in regulating mitochondrial function in aging intestinal stem cells [77]. In the present study, both *pten* and *asah2* were downregulated in the spleen and upregulated in the liver, indicating that these two genes may play distinct regulatory roles in these organs, which should be evaluated further.

*Tat*, a liver-expressed enzyme essential for tyrosine catabolism [78], is linked to metabolic disorders like hepatitis [79], diabetes [80], and obesity [81]. It also modulates tumor metabolism and invasiveness in cancers like hepatocellular carcinoma [82] and glioblastoma [83]. However, in PTC (papillary thyroid carcinoma), *tat* is downregulated, acting as a tumor suppressor, with low levels associated with invasion and metastasis [84]. Our study found *tat* downregulation too, hinting at its role in the tyrosine catabolism balance and metabolic processes under pathogen stress in fish.

In summary, *arg1*, *chmp1a*, *map2k6*, *chmp6*, *chmp2a*, *pten*, *tat*, and *asah2* are intricately involved in the metabolic processes of fish under pathogen stress, exerting significant effects on apoptosis, proliferation, and inflammatory responses. It is worth noting that these disease resistance-related genes were validated by examining expression levels at 72 h post-pathogen infection. Gene expression levels were not evaluated at other time points after infection and therefore some key genes may have been missed in the analysis. Future research should include analyses of gene expression levels at different time points post-infection, particularly for genes that did not show significant differences at 72 h, in order to further evaluate the molecular mechanisms underlying disease resistance.

## 4. Materials and Methods

### 4.1. Challenge Experiment and Sample Collection

The F1 full-sib family of small yellow croaker employed in this study were generated through artificial induction and fertilization methods, as described in Liu et al. [85]. Briefly, when the water temperature reached 15 °C, the one-year-old small yellow croaker were injected with luteinizing hormone releasing hormone A2 (Ningbo second hormone factory, Ningbo, China) at a dose of 5.0 μg kg^−1^ and 2.5 μg kg^−1^ body weight for female and male individuals, respectively, to stimulate spawning and spermatogenesis. Forty-eight hours after the injection, all the gonadal development of the fish were monitored every 2 h. Mature fish were utilized for constructing full-sib families through artificial fertilization. Fin tissue samples from the parental fish were collected and preserved in anhydrous ethanol (Anhui TEDIA High Purity Solvents Co., Ltd, Anqing, China). These F1 full-sib families were reared at Xiangshan Aquatic Company (Ningbo, China). After reaching the age of 8 months, a random selection of 121 individuals (22.72 ± 7.65 g body weight) from a single family were chosen for experimentation. Prior to the start of the experiment, the fish underwent a 2-week acclimation period in filtered seawater maintained at 18 °C, during which they were fed an artificial diet. Following acclimation, the fish were fasted for 24 h to standardize their physiological condition. Each fish was then administered an intraperitoneal injection of 0.5 mL of a *P. plecoglossicida* bacterial suspension, prepared at the 96 h median lethal concentration (1 × 10^3^ CFU/mL). The median lethal concentration was determined by conducting pre-infection tests at settings of 0, 1 × 10^2^, 1 × 10^3^, 1 × 10^4^, and 1 × 10^5^ CFU/mL concentrations. The survival time (phe_dur), measured from the time of injection to death, was recorded and utilized as an indicator of disease resistance. Tail fin samples were collected from all experimental fish and preserved in anhydrous ethanol for genomic DNA extraction.

### 4.2. Sequencing, Genotyping, and SNP Filtering

All fin samples underwent DNA extraction using the magnetic bead method. The concentration of extracted DNA was quantified using a Qubit fluorimeter (Invitrogen, Carlsbad, CA, USA), and DNA integrity was evaluated through 1% agarose gel electrophoresis. Only samples that met the quality control standards were used for the library construction for genotyping by pinpoint sequencing of liquid-captured targets. Following library construction, genotyping was performed using the small yellow croaker 100K liquid SNP chip, which included 100,031 SNPs [86]. The raw data underwent quality control using Fastp, and the resulting clean reads were aligned to the reference genome (GCA_040670005.1) using the BWA 0.7.17 [87] alignment software. Targeted SNPs were identified using the HaplotypeCaller tool from the GATK 4.0 [88] variant analysis software. The SNPs were then filtered based on the following criteria: parent depth > 20 and offspring depth > 3.

Loci with missing information for the parents were excluded. Subsequently, polymorphic loci between the parents were identified, and markers fitting the following segregation patterns were selected: lm × ll (1:1 segregation only in the female parent), nn × np (1:1 segregation only in the male parent), and hk × hk (1:2:1 segregation in both parents). After filtering the parental markers, the offspring genotypes at these polymorphic loci were discarded, with a minimum base support of ≥2. Alleles that were absent in the parents were removed, and markers that covered more than 85% of the offspring population were retained. To assess segregation distortion, chi-square tests were applied to the candidate markers with a significance threshold of *p* = 0.001. For the retained markers, the similarity between two adjacent markers were calculated based on their physical positions. If the similarity is greater than 0.9, they are grouped into a bin mark. Then, this bin mark is compared with the adjacent marker below. If the similarity is less than 0.9, it is divided into a new bin; otherwise, it is considered as a separate bin. Within each bin, the marker with the highest integrity was designated as the representative marker for that bin, ensuring the selection of the most reliable and informative markers for further analyses.

### 4.3. Genetic Map Construction and QTL Mapping

After filtering, high-quality genetic markers were utilized to construct a genetic linkage map using LepMap3 [89]. Linkage groups were assigned based on their chromosomal affiliation. Within each group, markers were ordered employing the maximum likelihood approach. The Kosambi [90] mapping function was then applied to compute the genetic distances between markers. An integrated map was constructed using male and female maps that were originally generated using a single input file. For visualization of the linkage map, the Perl SVG module was utilized.

For a QTL analysis of the phe_dur phenotypic data, the Mixed Quantitative Model (MQM) mapping method using MapQTL6.0 [91] was employed. A logarithm of the odds (LOD) score of 3 was set as the threshold to identify QTL intervals associated with the phe_dur trait. Candidate genes within these QTL intervals were pinpointed based on genome annotation data, with particular emphasis on genes in which the SNPs of interest were located, designating them as genes of heightened interest.

### 4.4. Gene Annotation and Enrichment Analysis

Based on the small yellow croaker reference genome, the functional annotation of genes located within the target QTL regions was conducted utilizing the Gene Ontology (GO, http://www.geneontology.org/, accessed on 29 September 2024) and Kyoto Encyclopedia of Genes and Genomes (KEGG, https://www.kegg.jp/, accessed on 29 September 2024) databases. Hypergeometric tests were employed to identify metabolic or signaling pathways that were significantly enriched among the candidate genes.

### 4.5. Analysis of Candidate Gene Expression Levels

A total of 120 healthy 8-month-old healthy small yellow croakers (21.81 ± 6.11 g body weight) were randomly selected and temporarily held in filtered seawater maintained at 18 °C for 2 weeks. Prior to the experiment, the fish were fasted for 24 h. Subsequently, the fish were divided into two groups (90 fish per group), test and control groups, subdivided into three replicates. The test group received an intraperitoneal injection of 0.5 mL of *P. plecoglossicida* at the median lethal dose (1 × 10^3^ CFU/mL), and the control group was injected intraperitoneally with 0.5 mL of TSB solution. After 72 h, spleen and liver tissues were collected from three fish per replicate in both the test and control groups. The collected tissue samples were immediately flash-frozen in liquid nitrogen and stored at −80 °C for RNA extraction.

To validate the expression changes of selected disease resistance-related genes in response to pathogen stimulation, total RNA was extracted using TRIzol™ Reagent (Thermo Fisher, Waltham, MA, USA), following the manufacturer’s procedure. RNA integrity was detected by Agilent 2100 (Santa Clara, CA, USA), and RNA integrity number > 7.0 was taken as the qualified standard. The quantity and quality of the total RNA were measured using a Nanodrop One spectrophotometer (NanoDrop, Waltham, MA, USA). Then they were reverse-transcribed into first-strand cDNA using the PrimeScript™ II 1st Strand cDNA Synthesis Kit (TaKaRa, Kusatsu, Japan). qRT-PCR was performed using the ABI 7500HT Real-time Detection System (Applied Biosystems, Waltham, MA, USA) and TransStart Tip Green qPCR SuperMix (TransGen Biotech, Beijing, China). PCR amplifications were performed in triplicate wells under standardized conditions: initial denaturation at 94 °C for 30 s followed by 40 cycles of denaturation at 94 °C for 5 s, annealing at 60 °C for 30 s, and extension at 72 °C for 10 s. The specific primers are detailed in Table 5. Each qPCR was replicated three times, and the expression data for each sample were normalized against β-actin levels using the 2^−ΔΔCT^ method [92]. The relative mRNA expression levels were analyzed using SPSS 22.0 (IBM, Armonk, NY, USA), and the figures were generated using GraphPad Prism9.

## 5. Conclusions

In this study, a 100K SNP chip was utilized for genotyping a full-sibling F1 family of small yellow croaker, yielding a high-density genetic map spanning 1395.72 cM with 0.08 cM intervals across 24 groups. Utilizing survival time post-challenge as the phenotypic trait, QTL mapping was performed, revealing 13 QTLs related to VWND resistance positioned on two linkage groups, explaining 10.9–13.3% of the phenotypic variation. These QTLs annotated 169 genes. Subsequently, a functional enrichment analysis was conducted, leading to the selection of 11 candidate genes pertinent to disease resistance. The expression levels of these genes were then measured in the spleen and liver of small yellow croaker infected with *P. plecoglossicida* using RT-qPCR. The results showed significant differential expression patterns in *arg1*, *chmp1a*, *map2k6*, *chmp2a*, *chmp6*, *pten*, *tat*, and *asah2*, indicative of their regulatory functions in response to disease resistance. Notably, this study represents the first effort in mapping QTLs associated with VWND resistance in the small yellow croaker, successfully pinpointing several functional genes that contribute to this disease resistance. As such, it lays the foundation for understanding disease resistance mechanisms in this species.

## Figures and Tables

**Table 1:** Types of markers. Proportion of each type of marker in the parental lines.

Marker Type	Female Genotype	Male Genotype	SNP Number	After Segregation	Bin Marker Number	Percentage
hkxhk	hk	hk	11,039	10,287	6112	22.60%
nnxnp	nn	np	18,052	15,912	6084	36.95%
lmxll	lm	ll	19,762	17,566	5840	40.45%
Total			48,853	43,765	18,036	100.00%

**Table 2:** Overview of genetic linkage groups. The map contained 17,735 bin markers, spanning a total genetic distance of 1395.72 cM, with an average distance of 0.08 cM and a maximum interval of 11.79 cM.

Linkage	Marker Number	Length	Average Distance	Max Gap
LG1	1202	59.93	0.05	3.73
LG2	1255	75.23	0.06	4.98
LG3	1125	65.73	0.06	4.97
LG4	734	49.19	0.07	4.98
LG5	690	61.60	0.09	4.14
LG6	837	58.75	0.07	7.92
LG7	796	44.67	0.06	4.56
LG8	657	62.40	0.09	2.07
LG9	370	91.09	0.25	7.92
LG10	715	57.04	0.08	3.73
LG11	994	55.79	0.06	1.65
LG12	980	69.87	0.07	4.56
LG13	364	54.61	0.15	7.49
LG14	697	64.07	0.09	4.14
LG15	357	30.17	0.08	2.89
LG16	852	64.95	0.08	7.92
LG17	547	53.72	0.10	2.07
LG18	1017	75.33	0.07	8.77
LG19	865	60.77	0.07	4.56
LG20	309	43.81	0.14	3.72
LG21	871	60.76	0.07	4.14
LG22	621	59.53	0.10	5.40
LG23	716	53.54	0.07	11.79
LG24	164	23.17	0.14	4.97
Total	17,735	1395.72	0.08	—

**Table 3:** Statistical information on disease resistance-related QTLs.

Group	Position	Peak Locus	Peak LOD	Expl (%)	Geno Number
9	22.37–24.02	hk2455	3.71	11–13.3	47
9	29.39	hk2465	3.16	11.4	1
9	30.22	hk2470	3.19	11.5	1
9	30.63	hk2461	3.03	11	0
21	2.48–3.31	np5073	3.09	10.9–11.2	4
21	3.31	hk5416	3.16	11.2–11.4	60
21	3.72	np5077	3.27	11.4–11.8	9
21	4.55	hk5428	3.03	11	1
21	4.55–6.2	hk5440	3.33	10.9–12	28
21	6.2	np5097	3.53	11.9–12.7	17
21	6.2	np5099	3.53	12–12.7	22
21	10.33	np5112	3.01	10.9	16
21	13.64–14.46	lm5172	3.06	10.9–11.1	16

**Table 4:** KEGG pathway analysis of 11 candidate genes.

Gene	Gene_id	KEGG Pathway
*chmp2a*	A022229	Endocytosis
*snf8*	A022226	Endocytosis
*chmp6*	A022157	Endocytosis
*chmp1a*	A009223	Endocytosis
*tat*	A009234	Phenylalanine, tyrosine and tryptophan biosynthesis; biosynthesis of amino acids; tyrosine metabolism
*asah2*	A022051	Sphingolipid metabolism
*pten*	A022010	Inositol phosphate metabolism
*arg1*	A022064	Arginine and proline metabolism; biosynthesis of amino acids
*heatr1*	A022065	Ribosome biogenesis in eukaryotes
*map2k6*	A022132	MAPK signaling pathway
*map3k14*	A022168	MAPK signaling pathway; intestinal immune network for IgA production

**Table 5:** Primer sequences. Primer sequences used for qRT-PCR verification.

Gene	Primer	Primer Sequences (5′–3′)
*chmp2a*	chmp2a-F	CGCTCAAGTCCAACAACAGC
	chmp2a-R	TCGATGGCGTCGTTCATCAT
*snf8*	snf8-F	CAGGACGTGAGCCAAGATGA
	snf8-R	CTCAGCCAGCTGCAGAACTA
*chmp6*	chmp6-F	CAGATCGGTAACCTGGAGCG
	chmp6-R	TCGATGGACATCACCTCGTG
*chmp1a*	chmp1a-F	GAAGGTCAAGAAGGCGTTGC
	chmp1a-R	GGCGGTCTGGACTTTAGAGG
*tat*	tat-F	TCAACGAGCTGTCCACCATC
	tat-R	CGCTGAAGCAGGAAGACAGA
*asah2*	asah2-F	CGAGTGGCACATTCCTCTGT
	asah2-R	GGCGACTTTGAAGACGTTGG
*pten*	Pten-F	TGTGCGGAACGACATTACGA
	Pten-R	TCCTCGCTCAACCACTTGTC
*arg1*	arg1-F	GATCCGTCACACAGGTCTCC
	arg1-R	TAATCCTGCGGGTGGTTTCC
*heatr1*	heatr1-F	CTCAGGCCGTTCAGGAAGTT
	heatr1-R	GTAGGTGGCGGCTTTGTAGT
*map2k6*	map2k6-F	AGATGTGAAGCCCTCCAACG
	map2k6-R	AGCCTTTCTGGTTCGTCTCG
*map3k14*	map3k14-F	TGCAGGGCGAATGTCTAAGG
	map3k14-R	GGAGCCACAGACAAGACTCC
*β-actin*	β-actin-F	CTCTGTCTGGATCGGAGGCT
	β-actin-R	GCTGAAGTTGTTGGGTGTTTG

## Data Availability

This whole-genome shotgun project of *L. polyactis* has been deposited in GenBank with accession number GCA_040670005.1.

## Supplementary Materials

The following supporting information can be downloaded at: https://www.mdpi.com/article/10.3390/ijms252010872/s1.

## References

[1] Chen Y., Wang W., Zhou W., Hu F., Wu M. (2022). Shifting Feeding Habits During Settlement Among Small Yellow Croakers (*Larimichthys polyactis*). Front. Mar. Sci..

[2] Wu T., Wu C., Fang Z., Ma X., Chen S., Hu Y. (2017). Effect of chitosan microcapsules loaded with nisin on the preservation of small yellow croaker. Food Control.

[3] Chen L., Zeng W., Rong Y., Lou B. (2021). Nutritional composition and textural quality of wild-caught and cage-cultured small yellow croaker (*Larimichthys polyactis*). J. Food Compos. Anal..

[4] Zhang Y., Yang F., Wang Z., You Q., Lou B., Xu D., Chen R., Zhan W., Liu F. (2017). Mitochondrial DNA variation and population genetic structure in the small yellow croaker at the coast of Yellow Sea and East China Sea. Biochem. Syst. Ecol..

[5] Liu F., Zhang T., He Y., Zhan W., Xie Q., Lou B. (2023). Integration of transcriptome and proteome analyses reveals the regulation mechanisms of *Larimichthys polyactis* liver exposed to heat stress. Fish Shellfish Immunol..

[6] Ina-Salwany M.Y., Al-Saari N., Mohamad A., Mursidi F.A., Mohd-Aris A., Amal M.N.A., Kasai H., Mino S., Sawabe T., Zamri-Saad M. (2019). Vibriosis in Fish: A Review on Disease Development and Prevention. J. Aquat. Anim. Health.

[7] Duan X., Li J., Shi H., Tao Z., Wei X., Ye Y., Guo B. (2024). Establishment of Nested PCR for the Detection of *Pseudomonas plecoglossicida* and Epidemiological Survey of *Larimichthys crocea* in the Southeast Coastal Region. Animals.

[8] Jiao J., Zhao L., Huang L., Qin Y., Su Y., Zheng W., Zhang J., Yan Q. (2021). The contributions of fliG gene to the pathogenicity of *Pseudomonas plecoglossicida* and pathogen-host interactions with *Epinephelus coioides*. Fish Shellfish Immunol..

[9] Akaylı T., Çanak Ö., Başaran B. (2011). A new Pseudomonas species observed in cultured young rainbow trout (*Oncorhynchus mykiss* Walbaum, 1792): *Pseudomonas plecoglossicida*. BIBAD.

[10] Yan L., Jin D., Yang S., Li X., Li H., Hu S., Sun Y., Yi G., Wang P., Rang J. (2022). Pathogenicity of fish pathogen *Pseudomonas plecoglossicida* and preparation of its inactivated vaccine. Microb. Pathog..

[11] Wang Y., Jin Y., Sun F., Zhang Y., Liu Q., Wang Q., Yang D., Zhang Y. (2023). The c-di-GMP signalling component YfiR regulates multiple bacterial phenotypes and virulence in Pseudomonas plecoglossicida. J. Appl. Microbiol..

[12] Zhang J., Zhou S., An S., Chen L., Wang G. (2014). Visceral granulomas in farmed large yellow croaker, *Larimichthys crocea* (Richardson), caused by a bacterial pathogen, *Pseudomonas plecoglossicida*. J. Fish. Dis..

[13] Hsu T.H., Chiu Y.T., Lee H.T., Gong H.Y., Huang C.W. (2021). Development of EST-Molecular Markers from RNA Sequencing for Genetic Management and Identification of Growth Traits in Potato Grouper (*Epinephelus tukula*). Biology.

[14] Uchino T., Tabata J., Yoshida K., Suzuki T., Noda T., Fujinami Y., Ozaki A. (2020). Novel Benedenia disease resistance QTLs in five F1 families of yellowtail (*Seriola quinqueradiata*). Aquaculture.

[15] Marana M.H., Karami A.M., Ødegård J., Zuo S., Jaafar R.M., Mathiessen H., von Gersdorff Jørgensen L., Kania P.W., Dalsgaard I., Nielsen T. (2021). Whole-genome association study searching for QTL for *Aeromonas salmonicida* resistance in rainbow trout. Sci. Rep..

[16] Grover A., Sharma P.C. (2016). Development and use of molecular markers: Past and present. Crit. Rev. Biotechnol..

[17] Robledo D., Palaiokostas C., Bargelloni L., Martínez P., Houston R. (2018). Applications of genotyping by sequencing in aquaculture breeding and genetics. Rev. Aquac..

[18] Tsai H.Y., Robledo D., Lowe N.R., Bekaert M., Taggart J.B., Bron J.E., Houston R.D. (2016). Construction and annotation of a high density SNP linkage map of the Atlantic Salmon (*Salmo salar*) genome. G3.

[19] Li Y., Liu S., Qin Z., Waldbieser G., Wang R., Sun L., Bao L., Danzmann R.G., Dunham R., Liu Z. (2015). Construction of a high-density, high-resolution genetic map and its integration with BAC-based physical map in channel catfish. DNA Res..

[20] Shao C., Niu Y., Rastas P., Liu Y., Xie Z., Li H., Wang L., Jiang Y., Tai S., Tian Y. (2015). Genome-wide SNP identification for the construction of a high-resolution genetic map of Japanese flounder (*Paralichthys olivaceus*): Applications to QTL mapping of *Vibrio anguillarum* disease resistance and comparative genomic analysis. DNA Res..

[21] Wang L., Wan Z.Y., Bai B., Huang S.Q., Chua E., Lee M., Pang H.Y., Wen Y.F., Liu P., Liu F. (2015). Construction of a high-density linkage map and fine mapping of QTL for growth in Asian seabass. Sci. Rep..

[22] Wang J., Li L., Zhang G. (2016). A high-density SNP genetic linkage map and QTL analysis of growth-related traits in a hybrid family of oysters (*Crassostrea gigas × Crassostrea angulata*) using genotyping-by-sequencing. G3.

[23] Xiao S., Wang P., Zhang Y., Fang L., Liu Y., Li J.T., Wang Z.Y. (2015). Gene map of large yellow croaker (*Larimichthys crocea*) provides insights into teleost genome evolution and conserved regions associated with growth. Sci. Rep..

[24] Weller C.A., Andreev I., Chambers M.J., Park M., Program N.C.S., Bloom J.S., Sadhu M.J. (2023). Highly complete long-read genomes reveal pangenomic variation underlying yeast phenotypic diversity. Genome Res..

[25] Sawayama E., Tanizawa S., Kitamura S.I., Nakayama K., Ohta K., Ozaki A., Takagi M. (2017). Identification of quantitative trait loci for resistance to RSIVD in red sea bream (*Pagrus major*). Mar. Biotechnol..

[26] Divilov K., Merz N., Schoolfield B., Green T., Langdon C. (2023). Marker-assisted selection in a Pacific oyster population for an antiviral QTL conferring increased survival to OsHV-1 mortality events in Tomales Bay. Aquaculture.

[27] Han M., Liu Y., Jin C., Wang X., Song W., He Y., Zhang Q. (2023). Potential loci and candidate genes associated with Vibrio anguillarum resistance in black rockfish (*Sebastes schlegelii*) revealed by BSA-seq analysis. Aquaculture.

[28] Rodríguez-Ramilo S.T., De La Herrán R., Ruiz-Rejón C., Hermida M., Fernández C., Pereiro P., Figueras A., Bouza C., Toro M.A., Martínez P. (2014). Identification of quantitative trait loci associated with resistance to viral haemorrhagic septicaemia (VHS) in turbot (*Scophthalmus maximus*): A comparison between bacterium, parasite and virus diseases. Mar. Biotechnol..

[29] Beck E.A., Currey M.C., Small C.M., Cresko W.A. (2020). QTL mapping of intestinal neutrophil variation in threespine stickleback reveals possible gene targets connecting intestinal inflammation and systemic health. G3.

[30] Wang L., Bai B., Huang S., Liu P., Wan Z.Y., Ye B., Wu J., Yue G.H. (2017). QTL mapping for resistance to Iridovirus in Asian Seabass using genotyping-by-sequencing. Mar. Biotechnol..

[31] Lu C., Laghari M.Y., Laghari M.Y., Zheng X., Cao D., Zhang X., Kuang Y., Li C., Cheng L., Mahboob S. (2017). Mapping quantitative trait loci and identifying candidate genes affecting feed conversion ratio based onto two linkage maps in common carp (*Cyprinus carpio* L). Aquaculture.

[32] Qiu C., Han Z., Li W., Ye K., Xie Y., Wang Z. (2018). A high-density genetic linkage map and QTL mapping for growth and sex of yellow drum (*Nibea albiflora*). Sci. Rep..

[33] Guo J., Wang A., Mao S., Xu X., Li J., Shen Y. (2021). Construction of high-density genetic linkage map and QTL mapping for growth performance in black carp (*Mylopharyngodon piceus*). Aquaculture.

[34] Guo J., Zhang M., Wang S., Xu X., Shen Y., Li J. (2022). A high-density genetic linkage map and QTL mapping for growth related traits in grass carp (*Ctenopharyngodon idella*). Aquaculture.

[35] Houston R.D. (2017). Future directions in breeding for disease resistance in aquaculture species. Rev. Bras. Zootec..

[36] Liu P., Wang L., Wan Z.Y., Ye B.Q., Huang S., Wong S.M., Yue G.H. (2016). Mapping QTL for Resistance Against Viral Nervous Necrosis Disease in Asian Seabass. Mar. Biotechnol..

[37] Wang X., Xu W., Liu Y., Wang L., Sun H., Chen S. (2016). Quantitative trait loci detection of *Edwardsiella tarda* resistance in Japanese flounder *Paralichthys olivaceus* using bulked segregant analysis. Chin. J. Oceanol. Limnol..

[38] Bajgar A., Kucerova K., Jonatova L., Tomcala A., Schneedorferova I., Okrouhlik J., Dolezal T. (2015). Extracellular adenosine mediates a systemic metabolic switch during immune response. PLoS Biol..

[39] Jeria E., Oyanedel D., Rojas R., Farlora R., Lira G., Mercado A., Muñoz K., Destoumieux-Garzón D., Brokordt K., Schmitt P. (2023). Resistance of *Argopecten purpuratus* scallop larvae to vibriosis is associated with the front-loading of immune genes and enhanced antimicrobial response. Front. Immunol..

[40] Zheng Z., Wang F., Aweya J.J., Li R., Yao D., Zhong M., Li S., Zhang Y. (2018). Comparative transcriptomic analysis of shrimp hemocytes in response to acute hepatopancreas necrosis disease (AHPND) causing *Vibrio parahemolyticus* infection. Fish Shellfish Immunol..

[41] Varghese T., Dasgupta S., Anand G., Rejish Kumar V.J., Sahu N.P., Pal A.K., Puthiyottil M. (2022). Dietary arginine attenuates hypoxia- induced HIF expression, metabolic responses and oxidative stress in Indian Major Carp, *Cirrhinus mrigala*. Comp. Biochem. Physiol. B Biochem. Mol. Biol..

[42] Chen Z., Ceballos-Francisco D., Guardiola F.A., Huang D., Esteban M.Á. (2020). Skin wound healing in gilthead seabream (*Sparus aurata* L.) fed diets supplemented with arginine. Fish Shellfish Immunol..

[43] Arlauckas S.P., Garren S.B., Garris C.S., Kohler R.H., Oh J., Pittet M.J., Weissleder R. (2018). Arg1 expression defines immunosuppressive subsets of tumor-associated macrophages. Theranostics.

[44] Lee B., Wu C.Y., Lin Y.W., Park S.W., Wei L.N. (2016). Synergistic activation of Arg1 gene by retinoic acid and IL-4 involves chromatin remodeling for transcription initiation and elongation coupling. Nucleic Acids Res..

[45] Bronte V., Zanovello P. (2005). Regulation of immune responses by L-arginine metabolism. Nat. Rev. Immunol..

[46] Rossetti I., Zambusi L., Finardi A., Bodini A., Provini L., Furlan R., Morara S. (2018). Calcitonin gene-related peptide decreases IL-1beta, IL-6 as well as Ym1, Arg1, CD163 expression in a brain tissue context-dependent manner while ameliorating experimental autoimmune encephalomyelitis. J. Neuroimmunol..

[47] Schmidt O., Teis D. (2012). The ESCRT machinery. Curr. Biol..

[48] Alonso Y., Adell M., Migliano S.M., Teis D. (2016). ESCRT-III and Vps4: A dynamic multipurpose tool for membrane budding and scission. FEBS J..

[49] Hurley J.H. (2015). ESCRTs are everywhere. EMBO J..

[50] Scanlon S.T. (2018). Fine-tuning pyroptosis with ESCRT-III. Science.

[51] Wu Y., Wu Y., Xu C., Sun W., You Z., Wang Y., Zhang S. (2022). CHMP1A suppresses the growth of renal cell carcinoma cells via regulation of the PI3K/mTOR/p53 signaling pathway. Genes Genom..

[52] Sun N., Zhang D., Ni N., Tang Z., Gao H., Ju Y., Dai X., Wang J., Gu P., Ji J. (2020). miR-17 regulates the proliferation and differentiation of retinal progenitor cells by targeting CHMP1A. Biochem. Biophys. Res. Commun..

[53] Sakamoto M., Shiiki T., Matsui S., Okamoto N., Koshimizu E., Tsuchida N., Uchiyama Y., Hamanaka K., Fujita A., Miyatake S. (2023). A novel homozygous CHMP1A variant arising from segmental uniparental disomy causes pontocerebellar hypoplasia type 8. J. Hum. Genet..

[54] You Z., Xin Y., Liu Y., Sun J., Zhou G., Gao H., Xu P., Chen Y., Chen G., Zhang L. (2012). Chmp1A acts as a tumor suppressor gene that inhibits proliferation of renal cell carcinoma. Cancer Lett..

[55] Hattori T., Takahashi Y., Chen L., Tang Z., Wills C.A., Liang X., Wang H.G. (2021). Targeting the ESCRT-III component CHMP2A for noncanonical Caspase-8 activation on autophagosomal membranes. Cell Death Differ..

[56] Bernareggi D., Xie Q., Prager B.C., Yun J., Cruz L.S., Pham T.V., Kim W., Lee X., Coffey M., Zalfa C. (2022). CHMP2A regulates tumor sensitivity to natural killer cell-mediated cytotoxicity. Nat. Commun..

[57] Fu D., Tian L., Peng Z., Deng W., Yuan J., Ma D., Shi T., Li D., Wang Y. (2009). Overexpression of CHMP6 induces cellular oncosis and apoptosis in HeLa cells. Biosci. Biotechnol. Biochem..

[58] Goliand I., Nachmias D., Gershony O., Elia N. (2014). Inhibition of ESCRT-II-CHMP6 interactions impedes cytokinetic abscission and leads to cell death. Mol. Biol. Cell.

[59] Yang S.H., Sharrocks A.D., Whitmarsh A.J. (2003). Transcriptional regulation by the MAP kinase signaling cascades. Gene.

[60] Zhang Q., Wang S., Zheng S., Zhang Z., Xu S. (2019). Chlorpyrifos Suppresses Neutrophil Extracellular Traps in Carp by Promoting Necroptosis and Inhibiting Respiratory Burst Caused by the PKC/MAPK Pathway. Oxid. Med. Cell. Longev..

[61] Chen J., Shao B., Wang J., Shen Z., Liu H., Li S. (2021). Chlorpyrifos caused necroptosis via MAPK/NF-κB/TNF-α pathway in common carp (*Cyprinus carpio* L.) gills. Comp. Biochem. Physiol. C Toxicol. Pharmacol..

[62] Deng W., Yang T., Dong R., Yan Y., Jiang Q. (2023). Astaxanthin protects tilapia head kidney cells against polystyrene microplastics-induced inflammation through MAPK and NF-κB signaling pathways. Aquaculture.

[63] Tao S., Li X., Wang J., Bai Y., Wang J., Yang Y., Zhao Z. (2024). Examination of the relationship of carbonate alkalinity stress and ammonia metabolism disorder-mediated apoptosis in the Chinese mitten crab, *Eriocheir sinensis*: Potential involvement of the ROS/MAPK signaling pathway. Aquaculture.

[64] Lotan T.L., Lyon M., Huo D., Taxy J.B., Brendler C., Foster B.A., Stadler W., Rinker-Schaeffer C.W. (2007). Up-regulation of MKK4, MKK6 and MKK7 during prostate cancer progression: An important role for SAPK signalling in prostatic neoplasia. J. Pathol..

[65] Tang C., Ou J., Kou L., Deng J., Luo S. (2020). Circ_016719 plays a critical role in neuron cell apoptosis induced by I/R via targeting miR-29c/Map2k6. Mol. Cell. Probes.

[66] Lin S., Liu K., Zhang Y., Jiang M., Lu R., Folts C.J., Gao X., Noble M.D., Zhao T., Zhou Z. (2018). Pharmacological targeting of p38 MAP-Kinase 6 (MAP2K6) inhibits the growth of esophageal adenocarcinoma. Cell Signal..

[67] Kumar V., Behera R., Lohite K., Karnik S., Kundu G.C. (2010). p38 kinase is crucial for osteopontin-induced furin expression that supports cervical cancer progression. Cancer Res..

[68] Lee Y.R., Chen M., Pandolfi P.P. (2018). The functions and regulation of the PTEN tumour suppressor: New modes and prospects. Nat. Rev. Mol. Cell Biol..

[69] Sun C., Tao Y., Gao Y., Xia Y., Liu Y., Wang G., Gu Y. (2018). F-box protein 11 promotes the growth and metastasis of gastric cancer via PI3K/AKT pathway-mediated EMT. Biomed. Pharmacother..

[70] Li W., Guan X., Sun L. (2020). hosphatase and Tensin Homolog (PTEN) of Japanese Flounder-Its Regulation by miRNA and Role in Autophagy, Apoptosis and Pathogen Infection. Int. J. Mol. Sci..

[71] Howe C., Mitchell J., Kim S.J., Im E., Rhee S.H. (2019). Pten gene deletion in intestinal epithelial cells enhances susceptibility to *Salmonella Typhimurium* infection in mice. J. Microbiol..

[72] Briercheck E.L., Trotta R., Chen L., Hartlage A.S., Cole J.P., Cole T.D., Mao C., Banerjee P.P., Hsu H.T., Mace E.M. (2015). PTEN is a negative regulator of NK cell cytolytic function. J. Immunol..

[73] Snider A.J., Wu B.X., Jenkins R.W., Sticca J.A., Kawamori T., Hannun Y.A., Obeid L.M. (2012). Loss of neutral ceramidase increases inflammation in a mouse model of inflammatory bowel disease. Prostaglandins Other Lipid Mediat..

[74] Maltesen H.R., Troelsen J.T., Olsen J. (2010). Identification of a functional hepatocyte nuclear factor 4 binding site in the neutral ceramidase promoter. J. Cell. Biochem..

[75] García-Barros M., Coant N., Kawamori T., Wada M., Snider A.J., Truman J.P., Wu B.X., Furuya H., Clarke C.J., Bialkowska A.B. (2016). Role of neutral ceramidase in colon cancer. FASEB J..

[76] Zhu H., Klement J.D., Lu C., Redd P.S., Yang D., Smith A.D., Poschel D.B., Zou J., Liu D., Wang P.G. (2021). Asah2 represses the p53-Hmox1 axis to protect myeloid-derived suppressor cells from ferroptosis. J. Immunol..

[77] Duan C., Wang Z., Wu J., Tan C., Fang F., Qian W., Han C., Hou X. (2024). Fut2 deficiency promotes intestinal stem cell aging by damaging mitochondrial functions via down-regulating α1,2-fucosylation of Asah2 and Npc1. Research.

[78] Wu Z., Zhang J., Jia Z., Yang Z., Liu S., Wang H., Zhao C., Zhao J., Tang Q., Xiong Y. (2024). TRIM21-mediated ubiquitylation of TAT suppresses liver metastasis in gallbladder cancer. Cancer Lett..

[79] Manzini B.M., da Silva Santos Duarte A., Sankaramanivel S., Ramos A.L., Latuf-Filho P., Escanhoela C., Kharmandayan P., Olalla Saad S.T., Boin I., Malheiros Luzo Â.C. (2015). Useful properties of undifferentiated mesenchymal stromal cells and adipose tissue as the source in liver-regenerative therapy studied in an animal model of severe acute fulminant hepatitis. Cytotherapy.

[80] Nandi S.S., Zheng H., Sharma N.M., Shahshahan H.R., Patel K.P., Mishra P.K. (2016). Lack of miR-133a decreases contractility of diabetic hearts: A role for novel cross talk between tyrosine aminotransferase and tyrosine hydroxylase. Diabetes.

[81] Adams S.H. (2011). Emerging perspectives on essential amino acid metabolism in obesity and the insulin-resistant state. Adv. Nutr..

[82] Sun L., Zhang L., Chen J., Li C., Sun H., Wang J., Xiao H. (2020). Activation of tyrosine metabolism in CD13+ cancer stem cells drives relapse in Hepatocellular Carcinoma. Cancer Res. Treat..

[83] Yamashita D., Bernstock J.D., Elsayed G., Sadahiro H., Mohyeldin A., Chagoya G., Ilyas A., Mooney J., Estevez-Ordonez D., Yamaguchi S. (2020). Targeting glioma-initiating cells via the tyrosine metabolic pathway. J. Neurosurg..

[84] Xie R., Lin J., Li W., Chen H., Zhang J., Zhong M., Xue J., Mo C., Chen L., Zhu Y. (2024). Homogentisic acid metabolism inhibits papillary thyroid carcinoma proliferation through ROS and p21-induced cell cycle arrest. Life Sci..

[85] Liu F., Liu Y., Chu T., Lou B., Zhan W., Chen R.Y. (2019). Interspecific hybridization and genetic characterization of *Larimichthys polyactis* (♀) and *L. crocea* (♂). Aquac. Int..

[86] Liu F., Ye T., Zhang T.L., Zhu J.J., Liu H.W., Li Q., Liu S.F., Guo D.D., Zhan W., Lou B. Development of a 100K SNP array derived from the high-quality genome of *Larimichthys polyactis* and its application in genomic selection for growth and disease resistance. Unpublished.

[87] Jung Y., Han D. (2022). BWA-MEME: BWA-MEM emulated with a machine learning approach. Bioinformatics.

[88] Brouard J.S., Bissonnette N. (2022). Variant calling from RNA-seq data using the GATK joint genotyping workflow. Methods Mol. Biol..

[89] Rastas P. (2017). Lep-MAP3: Robust linkage mapping even for low-coverage whole genome sequencing data. Bioinformatics.

[90] Vinod K.K. (2011). Kosambi and the genetic mapping function. Resonance.

[91] Van Ooijn J.W. (2009). Sofware for the Mapping of Quantitative Trait Loci in Experimental Populations of Diploid Species.

[92] Schmittgen T.D., Livak K.J. (2008). Analyzing real-time PCR data by the comparative C(T) method. Nat. Protoc..

